# Aortoesophageal fistulae following TEVAR: Case report and literature review

**DOI:** 10.1016/j.ijscr.2023.108126

**Published:** 2023-04-06

**Authors:** Carlos Eduardo Rey Chaves, Santiago Rojas, J.D Rosso, Mauricio Peláez, Elio Fabio Sánchez, Oscar Geovanny Hernández Rodríguez

**Affiliations:** a Cirugía General, Pontificia Universidad Javeriana, Facultad de Medicina, Hospital Universitario San Ignacio. Bogotá - Colombia; b Estudiante de posgrado Cirugía General, Pontificia Universidad Javeriana, Facultad de Medicina. Bogotá - Colombia; c Cirugía vascular periférica, Pontificia Universidad Javeriana, Facultad de Medicina, Hospital Universitario San Ignacio. Bogotá - Colombia; d Cirugía de Tórax, Pontificia Universidad Javeriana, Facultad de Medicina, Hospital Universitario San Ignacio. Bogotá - Colombia; e Cirugía General y Cirugía Oncológica. Coordinador Unidad Cirugía Oncológica. Profesor asistente Pontificia Universidad Javeriana, Facultad de Medicina, Hospital Universitario San Ignacio. Bogotá - Colombia

**Keywords:** Aortic disease, Esophageal diseases, TEVAR, Aortoesophageal fistulae

## Abstract

**Introduction:**

Aortoesophageal fistulae are an uncommon pathology, primarily due to the aortic pathology in more than 50 % of the cases, followed by foreign body ingestion, and advanced malignancies. Recently it is recognized after surgical management of thoracic aortic pathologies either open or endovascular, with increased rates of morbidity and mortality.

**Presentation of the case:**

We present a 62-year-old male patient with a previous history of thoracic endovascular aortic repair, who enters the emergency room with gastrointestinal bleeding and clinical signs of infection. Positive blood cultures, and tomographic signs include prosthetic gas, with endoscopic findings of aortoesophageal fistulae. Aggressive surgical management was performed including esophageal resection and gastrointestinal exclusion. Bleeding control was reached in the early postoperative period, nevertheless despite multidisciplinary management, the patient died 8 days after surgery.

**Clinical discussion:**

Aortoesophageal fistulae, remains to be an uncommon complication either of thoracic aortic aneurysm or after endovascular treatment of aortic aneurysm; with high rates of morbidity and mortality, should be suspected in every case with upper gastrointestinal bleeding in the context of a patient with aortic disease. Non-surgical management should be avoided due to the high risk of complications and mortality, aggressive management needs to be considered in each case according to clinical condition of the patient.

**Conclusion:**

Aortoesophageal fistulae remain an uncommon complication after TEVAR, with increased mortality and morbidity rates after complete treatment. Conservative management should be avoided to achieve bleeding control and prevent the extension of the infection.

## Highlights

• Artoesophageal fistula remains to be an uncommon complication after endovascular repair of aortic aneurysms.
• In the context of thoracic aortic aneurysm and upper gastrointestinal bleeding, aortoesophageal fistula should be suspected and ruled out before any surgical treatment.
• Aortoesophageal fistula remains to be a diagnostic and surgical challenge, requiring multidisciplinary management.

## 1 Introduction

Aortoesophageal fistulae (AF) are an uncommon complication with high rates of mortality even with complete management [1], [2]. Usually is secondary to aortic aneurysms with penetrating ulcers, trauma, foreign body ingestion, malignancies involving the esophagus or bronchogenic tissue, or thoracic open or endovascular procedures [1], [2], [3]. It is well known that primary aortoesophageal fistulae is due to the presence of thoracic aortic aneurysm in almost 50 % of the cases, and the diagnosis could be a challenge due to the variety of clinical presentation [1], [2], [3]. According to Svensson et al. [3], there is an incidence rate between 0,5 and 1,7 % of aortoesophageal fistulae following open aortic repairs [3]. In the last years since the introduction of Thoracic endovascular aortic repair (TEVAR) has become an alternative to open procedures with acceptable rates of morbidity and mortality, and is now the preferred approach in selected cases for patients with thoracic aortic pathologies, and according to some authors [1], [4] AF rates do not differ between the approaches (1.7–1.9 %).

Clinical presentations vary between patients and could be presented either with hemodynamically unstable patients due to massive bleeding, or with a chronic condition with hematemesis, fever, and clinical signs of infection [1], [2], [3], [4], [5]. There is a lack of evidence regarding this pathology due to the small prevalence, and most of the literature is in case reports and small series of cases, for that reason there is no standardized management [1], [2], [3], [4], [5], [6].

AF remains to be a diagnostic and therapeutic challenge for the surgeon, with increased rates of morbidity and mortality, treatment options are restricted and vary between conservative and aggressive surgical options [1], [4], and to date, there is only one case reported in Colombia [7]. The present paper aim it's to describe a clinical case of AF in the context of thoracic aortic disease, after ethical and institutional approval, previous informed consent was filled, following SCARE guidelines [8].

## 2 Presentation of the case

**Fig. 1:**
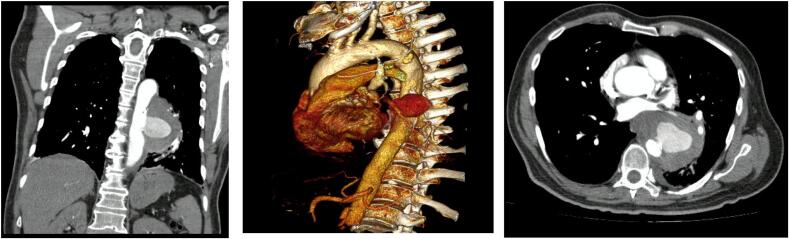
CT angiography with penetrating descending thoracic aortic ulcer contained between 4 and 5th Ishimaru zones.

**Fig. 2:**
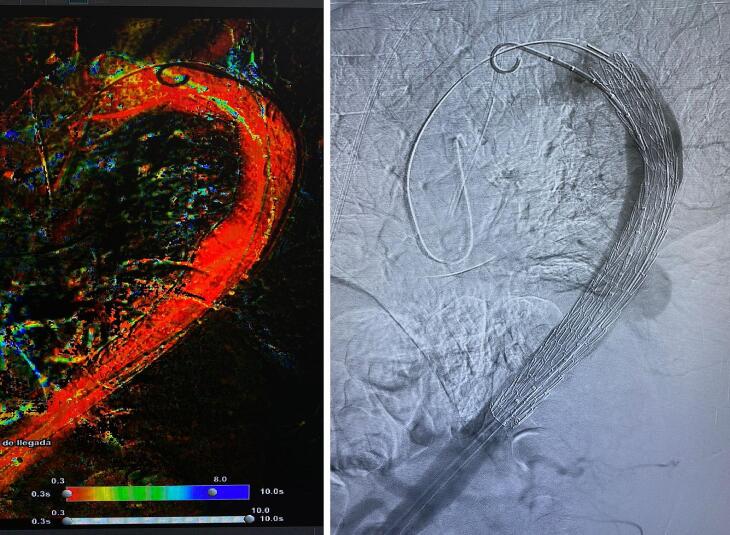
TEVAR.

We present a 62-year-old male patient who was consulted to the emergency room (ER) with a 15-day chest pain that didn't improve with non-steroidal anti-inflammatory drugs (NSAIDs) and hematemesis the day before the consult. Significant past medical history included type 2 diabetes and a heavy smoking habit. Initial workup revealed an elevated D dimer, which prompted a computed tomography (CT) angiography that demonstrated a penetrating descending aortic ulcer with secondary aneurysmal degeneration of 8 × 9 cm (Fig. 1). For that reason, he underwent a thoracic endovascular aortic repair (TEVAR) (Fig. 2). Esophagogastroduodenoscopy (EGD) does not evidence aortoesophageal fistulae at that moment, nevertheless, identifies gastric ulcers classified Forrest III.

He initially had an unremarkable postoperative course without spinal cord ischemia, new episodes of hematemesis, or any other symptom and was discharged at day 6.

**Fig. 3:**
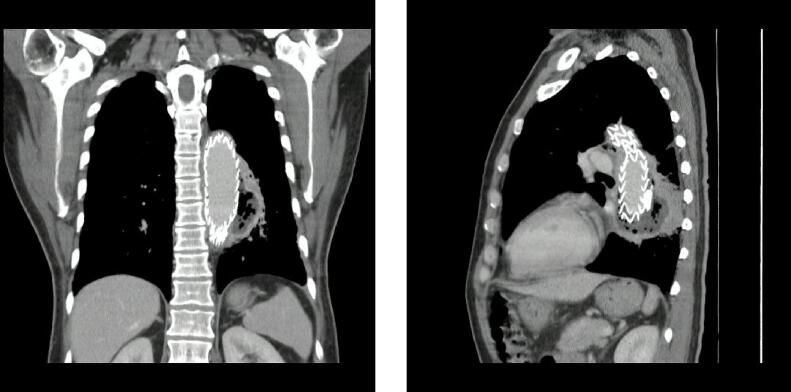
CT angiography with periprotesic gas suggestive of periprotesic infection.

**Fig. 4:**
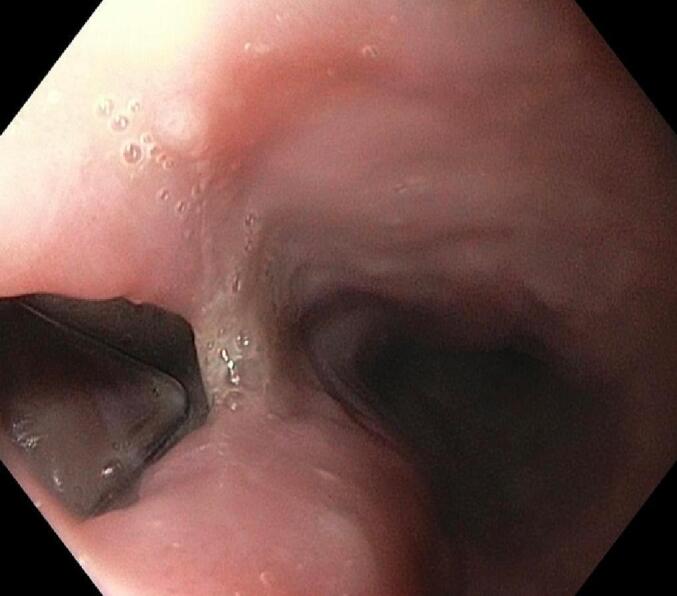
EGD with 12 mm aortoesophagic fistulae.

Eight days later he attended the ER with fever, diaphoresis, asthenia, and delusions, workup revealed positive blood cultures for gram-negative rods (*Escherichia coli* and Fusobacterium spp.). CT angiography was performed and evidenced gas surrounding the aortic prosthesis with the adequate exclusion of the aneurysm (Fig. 3) suggestive of periprosthetic infection. Broad-spectrum antibiotics were initiated and a new EGD was performed with a 12 mm aortoesophageal fistulae identified with aortic wall lysis and prosthetic exposition (Fig. 4). After the surgical joint including a vascular surgeon, thoracic surgeon, and gastroenterologist, he was taken to thoracoscopic and trans hiatal esophagectomy with cervical esophagostomy, jejunostomy, and decompressive gastrostomy. His postoperative course was complicated by difficult oral secretions management, dysphonia, and 5 days after surgery presented hemoptysis. He underwent a new CT angiography with the adequate exclusion of the aneurysm and without evidence of new fistulae. For that reason, a bronchoscopy evaluation was performed, showing signs of bleeding without a clear cause identified. After complete and multidisciplinary management, he died after seven days of surgery.

## 3 Discussion

Dubruelli in 1818 was the first one to describe this pathology [9]; later Hollander et al. [2] in their review identifies the most common causes of this complication and declares that in more than 50 % of the cases, are secondary to aortic complicated pathologies such as rupture of aortic aneurysm, followed by foreign body ingestion in approximately 20 % of the cases, and with comparable rates, advanced malignancies of the esophagus [2]. Some authors, such as Hooper et al. [1], [2], [3], [4], [5], [6], [7], [8], [9], documented a 9.4–20.4 % incidence of AF in associated with thoracic aortic aneurysm, in our case, there is a possibility that AF was present previous to the endovascular management of the aortic aneurysm, thus reflect the diagnostic challenge regarding this pathology, and must be suspected in cases with upper gastrointestinal bleeding associated with thoracic aortic aneurysm [1], [2], [3], [4], [5], [6], [7], [8], [9].

Nevertheless, AF is also evidenced as an uncommon complication after thoracic aortic repair either with an open or endovascular approach, with similar rates between them (1.7–1.9 %) according to Chiesa et al. [1]. Some studies report that presentation of this condition is not in all cases in the early postoperative period and could be diagnosed until 16 months according to Eggebrecht et al. [10], thus reflecting the complexity of the condition with a broad spectrum of presentation of this complication.

Diagnosis remains to be a challenge, due to the varied clinical presentation that includes in most cases upper gastrointestinal bleeding with or without hemodynamically instability [1], [10], however, some studies evidenced that could be presented with sepsis including clinical signs of infection, elevated biochemical inflammation markers, and positive blood cultures. In our case, the patient presented with this triad (gastrointestinal bleeding, clinical signs of sepsis, and positive blood cultures) in an early postoperative period after TEVAR. Thus, clinical signs must be recognized, and a high diagnosis suspicion of AF must be present.

In this clinical context, confirmation of the diagnosis needs to be done with CT angiography and with endoscopic evidence of the fistulae [6], [7], [10]. CT angiography is well recognized as the preferred imaging modality with 33–100 % of sensitivity and 40–90 % specificity and should be performed promptly with clinical suspicion [5], tomographic signs that definitely confirm the diagnosis is the ectopic gas adjacent to the aortic lumen or involving the prosthesis or total extravasation of the contrast fluid into the esophageal lumen, nevertheless is an uncommon finding [5], [10], other signs include thickening of the esophageal wall directly related with aortic wall or prosthesis, or hematoma surrounding the aortic wall near to the esophagus [10]. Endoscopic findings of the abnormal communication between the esophagus and the aortic tissue or with direct visualization of the stent graft confirm the diagnosis; CT and EGD should be performed promptly to perform an early diagnosis [10].

Management of this complication remains to be a challenge. According to the current evidence regarding the multiple case reports published [10], conservative treatment including broad-spectrum antibiotic therapy, proton pump inhibitors, and enteral exclusion with gastrostomy; nevertheless, this approach is related to fatal outcomes in all the cases according to the literature [10], thus data supports the surgical aggressive approach in this patients including esophageal resection, gastrointestinal exclusion and in some cases stent-graft exclusion for patients with an infected prosthesis [10]. Esophageal stenting may play a role in the emergency context to control massive hemorrhage and lead to more aggressive treatment, in fact, some reports show successful treatment of AF with esophageal stenting [6].

According to the present literature, aggressive surgical treatment focused on the management of the esophagus to avoid the progression of the infection and control the bleeding [1], [10], surgical options include the primary repair which is preferred only in selected cases with small perforations with no clinical signs of mediastinitis; and esophageal resection [10], simultaneous surgical management should be avoided, due to the increased risk of morbidity and postoperative mortality. The majority of the clinical reports show that patients treated with an aggressive surgical approach could be stabilized in the acute stage of the complication, however, in most cases, nutritional status and critically ill patients with concomitant systemic infection lead to irreversible damage to the esophageal tissue leading to fatal outcomes in the later postoperative period due to persistent bleeding, associated aorto-bronchial fistulae, and complicated systemic infection [10].

Our patient presented with a non- unstable gastrointestinal bleeding and systemic infection after TEVAR; aggressive surgical management was performed focused on the esophageal pathology, with esophageal resection and gastrointestinal exclusion, with initially controlling bleeding and infection; nevertheless, nutritional status and fragile condition leading to persistent bleeding, and after a multidisciplinary treatment, the patient finally died.

## 4 Conclusion

Aortoesophageal fistulae in the context of thoracic aortic disease and following TEVAR, remains to be an uncommon pathology, with increased mortality rates despite a multidisciplinary treatment. There isn't any standardized management for this complication due to the low incidence; however, according to the actual literature, conservative management should be avoided, and aggressive surgical management must be performed in order to control bleeding and prevent systemic infection. This case report contributes to increasing the evidence in the management of this pathology.

## Consent

Written informed consent was obtained from the patient for publication of this case report and accompanying images. A copy of the written consent is available for review by the Editor-in-Chief of this journal on request.

## Provenance and peer review

Not commissioned, externally peer reviewed.

## Ethical approval

Ethical approval was provided by the authors institution.

## Funding

This research did not receive any specific grant from the public, commercial, or not-for-profit funding agencies.

## Guarantor

Carlos Rey.

## Research registration number

None.

## CRediT authorship contribution statement

CR: Manuscript Writing, critical revision of the manuscript, data analysis.
SR: Data analysis, manuscript writing
JR: Data analysis, manuscript writing
MP: Manuscript Writing, critical revision of the manuscript, data analysis.
EFS: Manuscript Writing, critical revision of the manuscript, data analysis.
OHR: Manuscript Writing, critical revision of the manuscript, data analysis.

## Declaration of competing interest

Authors do not declare any conflict of interest.
